# Impact of Alirocumab on Release Markers of Atherosclerotic Plaque Vulnerability in Patients with Mixed Hyperlipidemia and Vulnerable Atherosclerotic Plaque

**DOI:** 10.3390/medicina58070969

**Published:** 2022-07-21

**Authors:** Michał Kosowski, Marcin Basiak, Marcin Hachuła, Bogusław Okopień

**Affiliations:** Department of Internal Medicine and Clinical Pharmacology, Medical University of Silesia, 40-752 Katowice, Poland; mbasiak@sum.edu.pl (M.B.); marcin.hachula@gmail.com (M.H.); bokopien@sum.edu.pl (B.O.)

**Keywords:** PCSK-9 inhibitors, atherosclerotic plaque, dyslipidemia, osteopontin, osteoprotegerin, metalloproteinase 2, metalloproteinase 9

## Abstract

*Background and Objectives*: Atherosclerosis is a disease in the pathogenesis of which plasma factors apart from elevated cholesterol levels play a keyrole. Such factors include osteopontin (OPN), osteoprotegerin (OPG), and metalloproteinases (MMPs), which are factors that may be responsible for the stabilization of atherosclerotic plaque. The aim of this study was to assess the effect of modern lipid-lowering therapy by using proprotein convertase subtilisin/kexin type 9 (PCSK-9) inhibitor on the concentrations of these factors. *Materials and Methods*: The study included people suffering from dyslipidemia who were eligible to start alirocumab therapy. In this group, the concentrations of OPN, OPG, and MMPs were assessed before the initiation of therapy and after three months of its duration. *Results:* In the study, we observed a statistically significant reduction in the concentrations of OPN, OPG (*p* < 0.001), and metalloproteinase 2 (MMP-2) (*p* < 0.05) after the applied therapy. Moreover, we noticed that in the group of patients soon to start alirocumab therapy, the concentrations of these factors were higher compared to the control group (*p* < 0.001). *Conclusions*: The results of our study show that therapy with alirocumab significantly reduces the concentration of factors that affect atherosclerotic plaque vulnerability, which may explain their important role in reducing cardiovascular risk in patients undergoing this therapy.

## 1. Introduction

Atherosclerosis is one of the most important and most common causes of cardiovascular disease, which, according to EUROSTAT data for 2016, has contributed to over one million deaths in European countries [1]. Very often, it also leads to stroke, myocardial infarction, and other peripheral artery diseases, which cause a significant increase in mortality in industrialized countries. Atherosclerotic plaque is formed by the deposition of lipids within the intima, activation of inflammatory cells, and increasing collagen synthesis in vascular smooth muscle cells [2]. Numerous studies show that the inflammatory process plays a crucial role in the formation of atherosclerotic plaque. This is reflected by the presence of a large number of inflammatory cells, mainly macrophages and T lymphocytes, within the atherosclerotic plaque [2,3]. Activated macrophages and T cells produce proinflammatory cytokines, hydrolysis enzymes, coagulation factors, and adhesion molecules that can be responsible for the progression and destabilization of atherosclerotic plaque [4,5,6]. High-risk or vulnerable plaques are associated with an increased risk of rupture, uncontrolled embolism, and vascular incidents. Changes in the histological structure of plaque show a large lipid core, a thin cover covered with fibrin, ulceration, the presence of wall clots or effusion inside, and intense infiltration of macrophages and other inflammatory cells [7]. As demonstrated by many authors, the majority of plaque ruptures are clinically silent [7,8,9]. Imaging methods, such as ultrasonography and the much more sensitive and specific magnetic resonance, are the most precise in detecting predictors of atherosclerotic destabilization. But these methods are expensive and not as practical as attempting to determine and describe plasma indicators of atherosclerotic destabilization. The role of biomarkers in detecting vulnerable plaque has not been fully understood and constitutes a wide field of research interest, both in the prevention of cardiovascular episodes and in the evaluation of the response to anti-atherosclerosis therapy [9,10]. Examples of these biomarkers are metalloproteinase 2 (MMP-2), metalloproteinase 9 (MMP-9), osteopontin (OPN), and osteoprotegerin (OPG).

Metalloproteinases (MMPs) are a family of endopeptidases responsible for the damage of the extracellular matrix (ECM). They are also involved in arterial remodeling processes [11]. Some of them, especially MMP-9 and MMP-2, are also involved in the process of atherogenesis. For this reason, we can observe higher concentrations of these MMPs in the endothelium of vessels undergoing atherogenesis [12]. A high concentration of MMP-9 is also linked to a higher risk of plaque rupture, according to many studies [13].

OPN is a phosphorylated glycoprotein found mainly in bone tissue and is responsible for the process of bone formation and calcification [14,15]. This protein is also known as a multifunctional protein, which is activated in many states of acute or chronic inflammation, for example in wound healing, autoimmune diseases, or fibrosis [16]. Studies show that OPN is also a component of atherosclerotic plaques responsible for their calcification process [17], and that there is a strong correlation between OPN mRNA expression within atherosclerotic plaques and the severity of the atherogenesis and calcification process [18]. Other studies also show that the severity of the narrowing of the arteries caused by atherosclerotic plaques is related to the level of OPN in the blood [19].

OPG is a glycoprotein belonging to the tumor necrosis factor α (TNFα) receptor family. It was originally discovered as a bone resorption inhibitor, and its expression and production are regulated by various cytokines and hormones [20]. In addition, OPG is produced in various types of organs such as the heart, arteries, veins, lungs, kidneys, and immune cells. It has also been proven to be expressed in coronary smooth muscle cells and the endothelium of blood vessels in vitro [21]. Protective effects of OPG supply on arterial calcification induced by warfarin and vitamin D intoxication have been observed in animal models [22]; in in vitro models, OPG prolonged endothelial cell survival by protecting them from apoptosis [23].

All of the above-mentioned cytokines play a major role in the formation and destabilization of atherosclerotic plaques. Because of this, they can be an important biomarker of the response to treatment for atherosclerosis (such as using proprotein convertase subtilisin/kexin type 9 (PCSK-9) inhibitors), especially since studies show that the use of statins leads to a significant decrease in MMPs, OPN, and OPG plasma concentration [24,25].

PCSK-9 inhibitors are a group of drugs registered in lipid-lowering therapy as an addition to statin and ezetimibe therapy in order to intensify this treatment and achieve the recommended therapeutic goals [26]. Apart from their main mechanism of action, they exert many additional pleiotropic effects, such as stabilization of atherosclerotic plaque, antineoplastic effects, or the ability to modify the course of bacterial infections, which are not described in the case of other drugs used in lipid-lowering therapy [27]. The undoubted advantage of using this group of drugs is also the much lower number of adverse effects, especially muscle symptoms, than in the case of statins [28]. It should be remembered, however, that due to the high cost of this therapy, it is not available to all patients and is recommended only in cases where standard lipid-lowering therapy is not effective.

The impact of modern lipid-lowering therapy using PCSK-9 inhibitors on MMPs, OPN, and OPG levels is also still not clear. The aim of our study was to assess whether one of the PCSK-9 inhibitors, alirocumab, has any effect on the concentration of markers of atherosclerotic plaque vulnerability in subjects with dyslipidemia.

## 2. Materials and Methods

Patients aged 18–75 years, diagnosed with asymptomatic atherosclerosis based on ultrasound common carotid intima-media thickness (CIMT) assessment, were included in the study.

The inclusion criteria were as follows: mixed hyperlipidemia (former Frederickson hyperlipidemia type 2B)—plasma total cholesterol (TC) > 200 mg/dL, low-density lipoprotein cholesterol (LDL) > 135 mg/dL, triglycerides (TG) > 150 mg/dL; CIMT > 1.5 mm; for women at least 24 months since the last menstruation, hysterectomy, ovarectomy, or use of hormonal contraception.

Patients were excluded from the study in the case of: other types of primary dyslipidemias; secondary dyslipidemias in the course of autoimmune disorders; thyroid diseases; chronic pancreatitis; nephrotic syndrome; liver and biliary tract diseases; obesity (body mass index > 30 kg/m^2^); alcoholism; any acute and chronic inflammatory processes; symptomatic congestive heart failure; unstable coronary artery disease; myocardial infarction or stroke within 6 months preceding the study; arterial hypertension; impaired renal or hepatic function, malabsorption syndromes, treatment with other hypolipidemic drugs within 3 months before the study (statins, fibrates, and ezetimibe); concomitant treatment with other drugs known to affect plasma lipid levels (i.e., polyunsaturated fatty acids, monacolin K); concomitant treatment with drugs that may affect inflammatory processes in the vascular wall (including nonsteroid anti-inflammatory drugs and angiotensin-converting enzyme inhibitors) within 3 months before the enrollment, ongoing hormonal replacement therapy or oral hormonal contraception.

Of the 118 screened patients, 21 fulfilled all the very detailed and narrow inclusion criteria and were eligible for study entry. All patients gave their written informed consent in accordance with the Declaration of Helsinki. The study protocol was approved by the Bioethical Committee of the Medical University of Silesia. Because lipid-lowering therapy is of proven value, the use of a placebo group was considered unethical.

All included individuals, complying throughout the entire study period with lifestyle modifications, were treated with a constant dose of alirocumab (150 mg), administered once every two weeks at the same time subcutaneously for 90 days.

Therefore, our control group included 14 age-, sex- and weight-matched healthy subjects.

Taking of the patients’ medical history, clinical examination, and venous blood sampling were all carried out before the start of therapy for evaluating safety laboratory parameters. Laboratory tests included total and differential blood cell count, blood sedimentation rate, creatine kinase, alanine and aspartate aminotransferases, gamma-glutamyltransferase, alkaline phosphatase, electrolytes, bilirubin, creatinine, total proteins, urine examination, glycated hemoglobin, and 12-lead electrocardiography.

Venous blood samples were collected after an overnight 12 h fast at 8 a.m. before the treatment. All the tests were carried out by a person blinded to the subject’s identity and all clinical details. Plasma lipids were assayed by routine laboratory techniques. LDL levels were measured directly. The plasma levels of MMP-2, MMP-9, OPN, and OPG were assessed by commercially available enzyme immunoassay methods using Cloud-Clone Corp., Houston, TX, USA; Diaclone, Besançon, France; and Bio-Vendor, Brno, Czech Republic kits as described by the manufacturer. All the laboratory tests were also performed in the control group. To avoid the freeze–thawing effect, each assay was performed on a single sample aliquot.

### Statistical Analysis

The data was processed using Statistica TIBCO Software Inc. (2017) version 13.3 software (Palo Alton, CA, USA), which was licensed to the Medical University of Silesia in Katowice. We used the Shapiro–Wilk test to assess the normality of distributions. To compare quantitative variables with normal distribution (concentration of OPG, MMP-9, TC, LDL, HDL, and non-high-density lipoprotein cholesterol (non-HDL), the *t*-test for dependent means was used. For dependent variables with abnormal distribution (concentration of OPN, MMP-2, and TG) we used the Wilcoxon test. The Mann–Whitney U test was used to compare independent variables due to the differences in the size of the study group and the control group. We also use Spearman’s rank correlation to assess the relationship between variables. We assumed a *p*-value of less than 0.05 was statistically significant.

## 3. Results

In the study group, no significant differences in terms of demographic data (age, gender, smoking, and weight) were observed. The characteristics of these two groups are presented in Table 1.

In the control group, statistically significantly lower concentrations of TC, LDL, non-HDL, and TG (*p* < 0.001), and higher concentrations of HDL (*p* < 0.05) were observed than in the study group before treatment. After therapy with PCSK-9 inhibitors, a statistically significant decrease in TC, LDL, non-HDL, and TG concentrations and an increase in HDL concentration were observed (*p* < 0.001) (Table 2).

We also observed that in the control group, concentrations of OPN, OPG, MMP-2, and MMP-9 (*p* < 0.001) are statistically significantly lower than in the study group before treatment.

**Figure 1:**
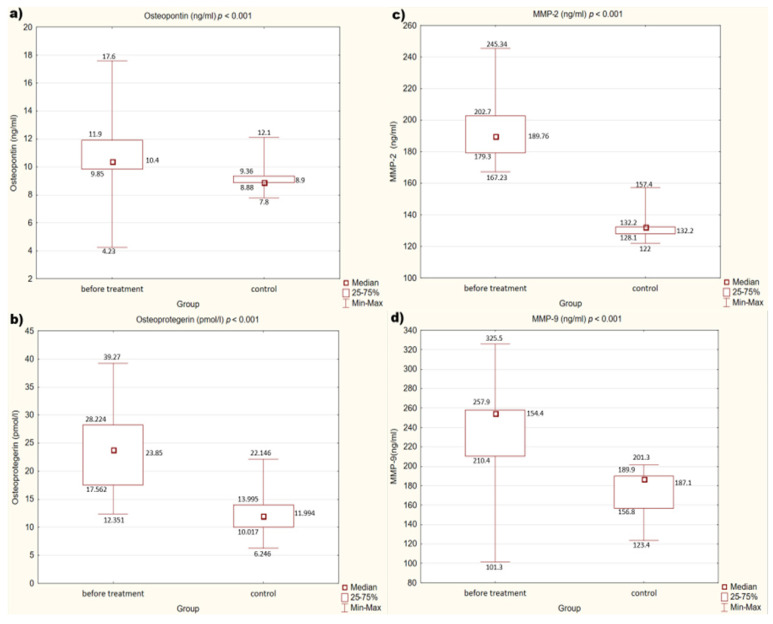
(**a**–**d**) Concentration of factors studied in the study and control groups. (**a**) Concentration of osteopontin (OPN) in study and control groups. (**b**) Concentration of osteoprotegerin (OPG) in study and control groups. (**c**) Concentration of metalloproteinase 2 (MMP-2) in study and control groups. (**d**) Concentration of metalloproteinase 9 (MMP-9) in study and control groups.

Detailed results are presented in Figure 1a–d.

We also assessed the effect of treatment with alirocumab on the concentrations of the individual mediators mentioned above. After treatment, a statistically significant decrease in concentrations of OPN, OPG (*p* < 0.001), and MMP-2 (*p* < 0.05) was observed. There are no statistically significant differences in MMP-9 concentrations before and after treatment (*p* = 0.11).

**Figure 2:**
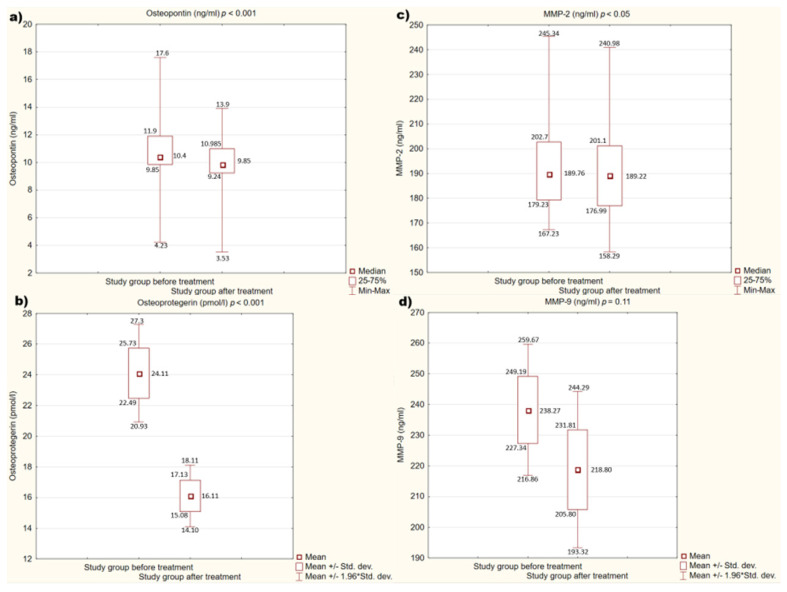
(**a**–**d**). Concentration of studied factors in the study group before and after treatment. (**a**) Concentration of osteopontin (OPN) in the study group before and after treatment. (**b**) Concentration of osteoprotegerin (OPG) in the study group before and after treatment. (**c**) Concentration of metalloproteinase 2 (MMP-2) in the study group before and after treatment. (**d**) Concentration of metalloproteinase 9 (MMP-9) in the study group before and after treatment.

Figure 2a–d present the detailed results.

We also checked the relationship between the examined factors affecting atherosclerotic plaque and the concentrations of cholesterol fractions. However, both in the group before and after treatment, no statistically significant correlation between these factors was observed. There was also no relationship between the reduction in the concentration of cholesterol fractions and the cytokines tested (*p* > 0.05). 

## 4. Discussion

A generous panel of cytokines is involved in the pathogenesis of atherosclerosis, including both OPN and OPG. Both of these proteins seem to play a key role in the calcification of vessel walls and atherosclerotic plaque calcification, which is related to many similarities between the bone formation process and the calcification of the vascular wall [29,30,31]. In addition, the OPN is involved in the synthesis of MMPs, which, by destroying ECM, are also involved in the process of damaging atherosclerotic plaques [32,33].

Due to the described mechanism of action, the relationship between the concentrations of MMPs, OPN, and OPG and the risk of cardiovascular disease (CVD) events in patients has been studied for many years. As the research shows, all these factors contribute to the increased CVD risk in patients [34,35,36,37]. These observations are consistent with the results of our study, in which in the control group, which included healthy people, the concentrations of MMPs, OPN, and OPG were significantly lower than in the group of people diagnosed with atherosclerosis.

Because of the complicated pathogenesis of the atherosclerotic process, studies were conducted at the turn of the century to document the impact of the previously widely used lipid-lowering therapy with statins on additional factors influencing CVD risk. It has been proven that this therapy affects not only the concentration of cholesterol but also the concentration of pro-inflammatory factors, and thus causes a greater reduction of CVD risk than results from only lowering the concentration of LDL [38,39]. In addition, other studies conducted in humans and in animal models have also shown that statin therapy significantly lowers the levels of OPN, OPG, MMP-2, and MMP-9 [40,41,42,43]. These actions stabilize the atherosclerotic plaque, thus reducing the CVD risk.

More importantly, in animal models, the relationship between the concentration of PCSK-9 and calcification markers, such as OPG itself, has been proven [44], although such a relationship has not been confirmed in studies on patients requiring hemodialysis [45]. In the case of OPN, in a study by Lupo et al., it was clearly found that a lower concentration of PCSK-9 is associated with a lower concentration of calcification markers such as OPN [46]. Data on the relationship between their concentration and PCSK-9 concentration is currently lacking.

These data prompted researchers to check whether modern lipid-lowering therapies, e.g., using PCSK-9 inhibitors, also carry such beneficial pleiotropic effects, especially given that studies such as CANTOS or FOURIER have shown that the CVD risk during treatment with these drugs decreases disproportionately to the lowering of LDL concentration [47].

It is important that studies using intravascular ultrasound (IVUS) have shown a significant relationship between the concentration of PCSK-9 in atherosclerotic plaque and the size of the necrotic core, which was independent of the LDL concentration [48]. Furthermore, studies conducted in 2020 which assessed atherosclerotic plaques in the same way indicated the positive effect of therapy with PCSK-9 inhibitors on reducing the lipid core burden index, which is one of the most important indicators of its stabilization [49].

To the best of our knowledge, no studies have been carried out so far that would show the effect of PCSK-9 inhibitors on the concentrations of OPN, OPG, and MMPs. Only one study in an animal model by Elsweidy M.M et al. found that policosanol, a drug that is capable of lowering PCSK-9 concentration, can reduce OPN concentration in atherosclerotic plaques [50]. This observation is consistent with our results, which confirm a reduction in the concentration of OPN, but also OPG and MMP-2 after the use of drugs that inhibit the action of PCSK-9. Moreover, in one of the studies conducted by Otake H., which included patients treated with alirocumab, plasma stabilization factors for atherosclerotic plaque were determined. However, the authors do not describe in this study the influence of alirocumab on these factors, but only describe the positive effect of this therapy on the plaque vulnerability assessed by optical coherence tomography (OCT) [51].

Furthermore, an important observation in our study is a statistically significant reduction in triglyceride levels in patients treated with alirocumab. This observation is not consistent with the studies carried out so far [52,53], but it may be due to the fact that the study group included people with newly diagnosed dyslipidemia who had not been treated with any lipid-lowering or dietary therapy so far. The initiation of dietary treatment could, in this case, lead to a decrease in triglyceride levels [54].

A major limitation of our study is the small sample size. The study was short in duration and did not assess clinical outcomes, and the study population, although exceeding the required sample size, was relatively low. However, it results from a relatively small number of patients treated with PCSK-9 inhibitors in our country. The second limitation is the lack of a placebo group. However, this is due to the fact that postponing treatment with a PCSK-9 inhibitor in this group of patients would be unethical. Also, the lack of ultrasound control after treatment is a significant limitation, as it does not allow us to assess the effect of treatment on the atherosclerotic plaque itself. The relatively short follow-up time of only 3 months may also be an important limitation. For this reason, we believe that additional studies are needed on a larger group of patients, which will clearly define the effect of treatment with PCSK-9 inhibitors on the concentration of MMPs, OPN, and OPG.

## 5. Conclusions

Therapy with alirocumab reduces the concentration of MMPs, OPN, and OPG, which are factors that may be responsible for the stabilization of atherosclerotic plaque. Additional studies are needed to unequivocally assess the impact of modern lipid-lowering therapy on the levels of the above-mentioned markers.

## Figures and Tables

**Table 1:** Baseline characteristics of patients (values are mean ± standard deviation (SD) unless indicated otherwise).

	Control Group	Study Group
Number of patients	14	21
Age, years	45 ± 5	47 ± 5
Women, %	35	34
BMI	27.5 ± 2.6	28.1 ± 2.2
Smokers, %	22	24
Systolic blood pressure, mmHg	133 ± 5	133 ± 6
Diastolic blood pressure, mmHg	82 ± 4	84 ± 4
Total cholesterol, mg/dL	163.4 ± 17.7	250.6 ± 35.4
LDL cholesterol, mg/dL	93.5 ± 15.9	172.7 ± 30
HDL cholesterol, mg/dL	46.3 ± 5.1	40.3 ± 10.1
Triglycerides, mg/dL	121.1 ± 21.1	178.4 ± 20.4
Fasting glucose, mg/dL	91 ± 6	92 ± 5

**Table 2:** Effect of alirocumab on plasma lipids.

	Control			Study Group before Treatment			Study Group after Treatment			
				Mean	SD		Mean	SD		* p *
TC (mg/dL)				250.6	35.4		173.8	31.6		<0.001 **
LDL (mg/dL)				172.7	30		94.4	29.1		<0.001 **
HDL (mg/dL)				40.3	10.1		49.5	10.3		<0.001 **
non-HDL (mg/dL)				210.3	34.2		124.3	34.9		<0.001 **
	** Median **	** Q1 **	** Q3 **	** Median **	** Q1 **	** Q3 **	** Median **	** Q1 **	** Q3 **	
TG (mg/dL)	121.1	99.8	142.2	178.4	168	198.8	142.8	123.8	157.1	<0.001 *, <0.001 **
TC (mg/dL)	168.2	151.1	174.6	246.2	224.7	278.2				<0.001 *
LDL (mg/dL)	94.7	89.8	105.6	167.6	155.5	201.4				<0.001 *
HDL (mg/dL)	47.1	41.1	48.3	41	34.2	43.1				<0.05 *
non-HDL (mg/dL)	121.1	105.4	133.2	203.2	188.3	246.1				<0.001 *

TC—total cholesterol; LDL—low-density lipoprotein cholesterol; HDL—high-density lipoprotein cholesterol; non-HDL—non-high-density lipoprotein cholesterol; TG—triglycerides; SD—standard deviation; Q1—first quartile; Q3—third quartile * *p*-value for study group before treatment versus control; ** *p*-value for study group before treatment versus study group after treatment.

## Data Availability

The data that support the findings of this study are available from the corresponding author (mbasiak@sum.edu.pl) on reasonable request.
